# Pentoxifylline immunomodulation in the treatment of experimental chronic pulmonary paracoccidioidomycosis

**DOI:** 10.1186/s13069-015-0027-8

**Published:** 2015-06-01

**Authors:** Damaris Elena Lopera, Tonny Williams Naranjo, José Miguel Hidalgo, Laura Echeverri, Jairo Hernando Patiño, Ángela Restrepo Moreno, Henrique Leonel Lenzi, Luz Elena Cano

**Affiliations:** Medical and Experimental Mycology Group, Corporación para Investigaciones Biológicas, Medellín, Colombia; Escuela de Ciencias de la Salud, Universidad Pontificia Bolivariana, Medellín, Colombia; Department of Radiology, Hospital Universitario San Vicente de Paúl, Medellín, Colombia; Laboratory of Pathology, Instituto Oswaldo Cruz, Fundação Oswaldo Cruz, Rio de Janeiro, Brazil; Microbiology School, Universidad de Antioquia, Medellín, Colombia

**Keywords:** Paracoccidioidomycosis, Lung fibrosis, Pentoxifylline

## Abstract

**Background:**

Pentoxifylline (PTX) is a methylxanthine compound with immunomodulatory and antifibrotic properties. The simultaneous use of PTX and antifungal therapy (itraconazole) has previously been evaluated in an experimental model of pulmonary paracoccidioidomycosis (PCM), a systemic fungal disease caused by the fungus *Paracoccidioides brasiliensis* (*Pb*) and characterized by chronic inflammation and lung fibrosis that appears even after a successful course of antifungal therapy. The results revealed prompt and statistically significant reductions in inflammation and fibrosis when compared to itraconazole alone. However, the effect of monotherapy with PTX on the host response to PCM has not been well-documented. Our aim was to determine the effect of PTX on the course of pulmonary lesions and on the local immune response.

**Results:**

At the middle and end of treatment, the *Pb*-infected-PTX-treated mice exhibited significant reductions in lung density compared to the *Pb*-infected-non-treated mice as assessed by the quantification of Hounsfield units on high-resolution computed tomography (HRCT) (*p <*0.05 by Kruskal-Wallis test); additionally, at the end of therapy, the lung areas involved in the inflammatory reactions were only 3 vs. 22 %, respectively, by histomorphometry (*p <*0.05 by Mann–Whitney test), and this reduction was associated with a lower fungal burden and limited collagen increment in the pulmonary lesions. PTX treatment restored the levels of IFN-γ, MIP-1β, and IL-3 that had been down-regulated by *Pb* infection. Additionally, IL-12p70, IL-10, IL-13, and eotaxin were significantly increased, whereas Regulated upon Activation, Normal T cell Expressed and Secreted (RANTES) levels were decreased in the lungs of the *Pb*-infected-PTX-treated mice compared to the non-treated group.

**Conclusions/significance:**

This study showed that PTX therapy administered at an “early” stage of granulomatous inflammation controlled the progress of the PCM by diminishing the pulmonary inflammation and the fungal burden and avoiding the appearance of collagen deposits in the pulmonary lesions.

## Background

Pentoxifylline (PTX) is one of the several methylxanthine compounds that have immunomodulatory properties. PTX acts as cAMP-phosphodiesterases inhibitor and exerts its cellular effects on erythrocytes, platelets, endothelial cells, PMNs, macrophages, and fibroblasts [1]. PTX reduces the production of inflammatory cytokines, such as TNF, IL-1α, IL-6, and IL-8, by phagocytes [2] and prevents their subsequent effects, such as leukocyte adherence, migration, and degranulation [3]. Additionally, PTX has antifibrotic properties; it exerts an antiproliferative effect on fibroblasts and inhibits extracellular matrix synthesis [4, 5].

Because inflammation participates in the pathogenesis and progression of many diseases, the therapeutic use of PTX has been studied alone or as an adjuvant therapy in different conditions, including infectious processes, and has produced positive effects. PTX prevents murine cerebral malaria [6] and improves the prognosis of this disease in humans [7, 8], decreases leukocyte recruitment into the cerebrospinal fluid in experimental bacterial meningitis [9], decreases organ damage, and improves survival in humans [10, 11] and in animals with sepsis [12]. Additionally, PTX has been used simultaneously with amphotericin-B to improve survival and to decrease fungal burden in cerebral cryptococcosis [13].

In a previous study, we evaluated a combined PTX and itraconazole therapy in a chronic experimental model of paracoccidioidomycosis (PCM) [14], which is a systemic mycosis produced by the thermally dimorphic fungus *Paracoccidioides brasiliensis* (*Pb*) that induces granulomatous inflammation and frequently progresses to pulmonary fibrosis [14, 15]. In that study, we reported prompt reductions of pulmonary granulomatous inflammation and fibrosis when compared to itraconazole treatment alone [14]. However, the effect of monotherapy with PTX on the host response to PCM has not been well-documented.

To better understand the effect of the immunomodulatory therapy with PTX on the course of fungal pulmonary lesions, we used an experimental mouse model of PCM to determine the local immune response and compared the lungs of treated versus non-treated *Pb*-infected mice with high-resolution computed tomography (HRCT), histopathology/histomorphometry, and assessments of the levels of cytokines, chemokines, and growth factors.

## Results

### PTX decreased the pulmonary density in the *Pb*-infected mice

**Fig. 1:**
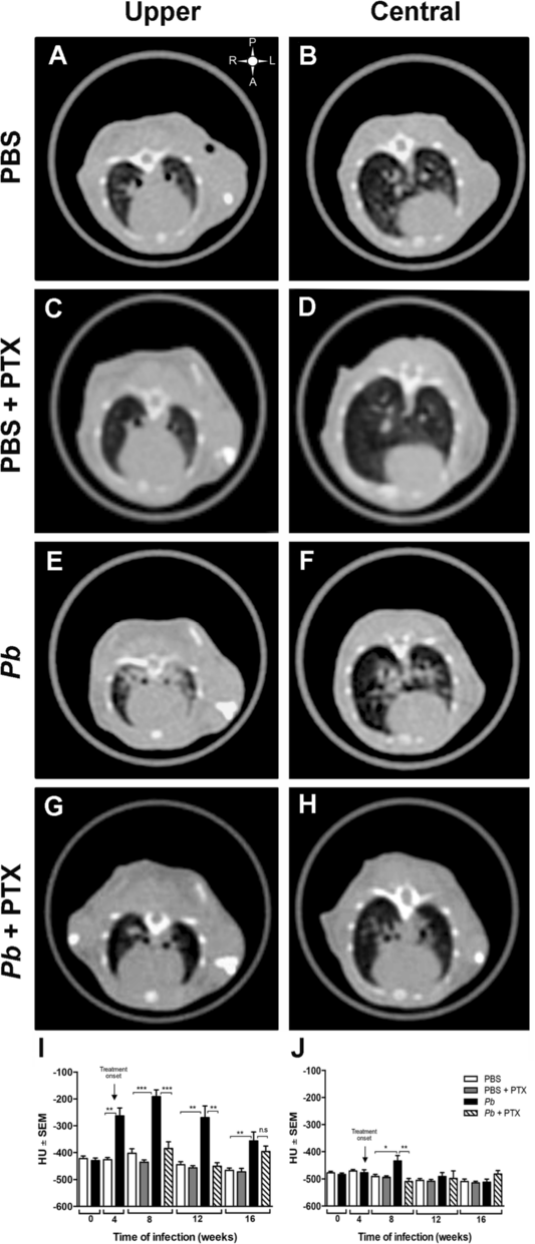
PTX treatment decreases pulmonary density in *Pb*-infected mice. Representative HRCT images of upper and central pulmonary regions obtained from PBS-inoculated mice (**a**, **b**), PBS-inoculated mice treated with PTX (**c**, **d**), *Pb*-infected mice (**e**, **f**), and *Pb*-infected mice treated with PTX (**g**, **h**) at 12 weeks. Pulmonary density was measured in the lung parenchyma as described in the “Methods” section and is expressed as the mean of Hounsfield units (HU) ± SEM. **i**, **j** correspond to HUs measured in the upper (I) and central (J) lung regions at 0, 4, 8, 12, and 16 weeks. The upper right symbol in (**a**) gives the spatial position of posterior (*P*), anterior (*A*), left (*L*), and right (*R*) regions. *n* = 10 mice per group at each period of evaluation, *p <0.05; **p <0.01; ***p <0.001*. ns* not significant

Before treatment (week 4), the *Pb-*infected mice exhibited peribronchial consolidations that were associated with a significant increase in the upper lung densities (−263 ± 29 vs. −426 ± 68 Hounsfield units (HU) in the control mice, *p <* 0.001*)*. These increases in density were significantly different compared to the control at all periods of evaluation (Fig. 1a, e, i). The pulmonary lesions tended to extend to the central region of the lung, but the increase in lung density was only higher at week 8 (Fig. 1f,j). After 4 weeks of PTX treatment (8 weeks post-infection (p.i)), the peribronchial consolidations decreased and were present in 4/10 infected treated mice (agreement between the radiologists (*κ*) = 0.8) in contrast to 10/10 mice in the *Pb*-infected group (*κ* = 1.0). Although multiple nodules were reported for both groups, the agreement among the radiologists at this time was low (*κ* = 0.4). At the end of therapy (12 weeks p.i), marked differences between the groups were noticeable (Fig. 1e,g). The infected mice continued to show the same pattern of lesions, but they tended to be more severe. Indeed, at this time, one of the infected animals also presented with atelectasis and another presented with pleural thickness. In contrast, 8/10 of the mice that were treated for 8 weeks with PTX exhibited only small opacities that were referred to as unique nodules (Fig. 1g) accompanied by significant overall reductions in pulmonary density compared with the *Pb*-infected non-treated mice (−451 ± 68 vs. −269 ± 25 HU; *p <* 0.001). However, 4 weeks post-treatment, the pulmonary densities were reactivated in all animals (*κ* = 1; Fig. 1i) and diffused bronchial dilatation was even observed.

The administration of PTX to the healthy non-infected mice did not induce changes in the normal appearance of the lungs or modify the pulmonary densities as expressed in HU at any observation time (Fig. 1c,d,i,j).

### PTX decreased the pulmonary inflammation area in the *Pb*-infected mice

**Fig. 2:**
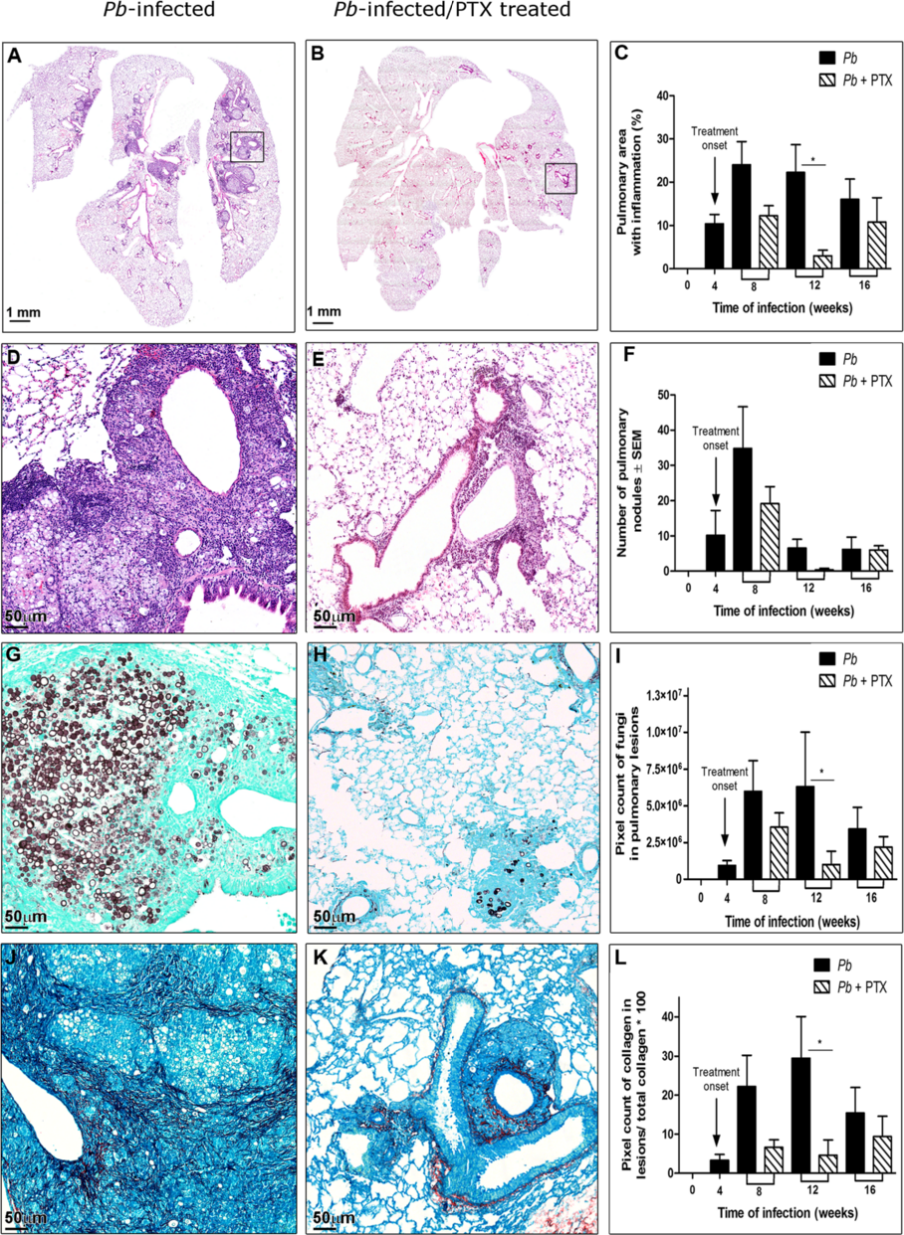
PTX treatment significantly reduces pulmonary inflammation and fungal burden and hinders collagen deposition in *Pb*-infected treated mice. **a**, **d**, **g**, **j** correspond to photomicrographs of lung sections of a representative *Pb*-infected mouse at 12 weeks p.i. stained with H&E, Grocott, and PIFG, respectively. **b**, **e**, **h**, **k** correspond to photomicrographs of lung sections of a representative *Pb*-infected mouse, treated with PTX at the end of the therapy (12 weeks p.i) stained with H&E, Grocott, and PIFG, respectively. **c**, **f**, **i**, **l** are histomorphometric quantification graphs of inflammation, nodules, fungal burden, and collagen deposition in the pulmonary lesions of *Pb*-infected mice treated or not with PTX. *n* = 5 mice per group at each period of evaluation, *p <0.05

Before the beginning of the PTX treatment at 4 weeks p.i, the *Pb*-infected mice presented pulmonary nodules and evidence of incipient periarterial inflammation accompanied by dispersed and intense parenchymal inflammation. In the absence of PTX treatment, these histopathological changes remained present and increased to reach their greatest intensities at 8 and 12 weeks p.i. Four weeks after the treatment onset (8 weeks p.i), the lung area occupied by inflammatory reactions was reduced compared to the *Pb*-infected group, but this difference was only statistically significant at the end of the treatment (12 weeks) when the average lung inflammation diminished to 3 ± 2 % vs. 22 ± 7 % in the non-treated group (*p <*0.05) (Fig. 2a–c). In the middle of the therapy, PTX decreased the dispersed parenchymal inflammation and the cellularity at the outer regions of the nodules, which prevented their confluence. At the end of the treatment, only two nodules were observed in five lungs (Fig. 2d–f), and only small focal areas of periarterial inflammation composed of lymphoplasmocytic infiltrate remained in the lungs (Fig. 2e).

### PTX decreased the fungal burden in the lungs of the *Pb*-infected mice

In the *Pb*-infected mice, the morphologies and quantities of the fungi in the lung tissues varied depending on the locations of the lesions and the time of the infection. There were trends toward increases at 8 and 12 weeks p.i. In the nodules, the fungal cells were restricted to the central zone, and large yeasts predominated (10–20 μm; Fig. 2g). In the perivascular lesions, the fungi were more dispersed and frequently presented with perifungal spaces surrounding large mother cells with multiple small buds of less than 2 μm.

The PTX-treated mice exhibited lesser amounts of *Pb* yeast in the pulmonary lesions as evidenced by pixel counting of the stained yeast, but some small buds, mainly in the periarterial regions, persisted (Fig. 2h,i).

### PTX prevented collagen increments in the pulmonary lesions

In the *Pb*-infected mice, the collagen fibers gradually increased up to 12 weeks in parallel with the inflammation. The collagen depositions arranged themselves as pseudocapsule around the nodules and were more prominent at the periarterial lesions (Fig. 2j). Measurements of the collagen pixels inside the lesions and comparisons between groups revealed that the PTX-treated mice did not exhibit increases in collagen in the pulmonary lesions while the *Pb*-infected untreated mice did (Fig. 2k,l).

### Immunomodulatory effect of PTX at the lung level in healthy and *Pb*-infected mice

**Fig. 3:**
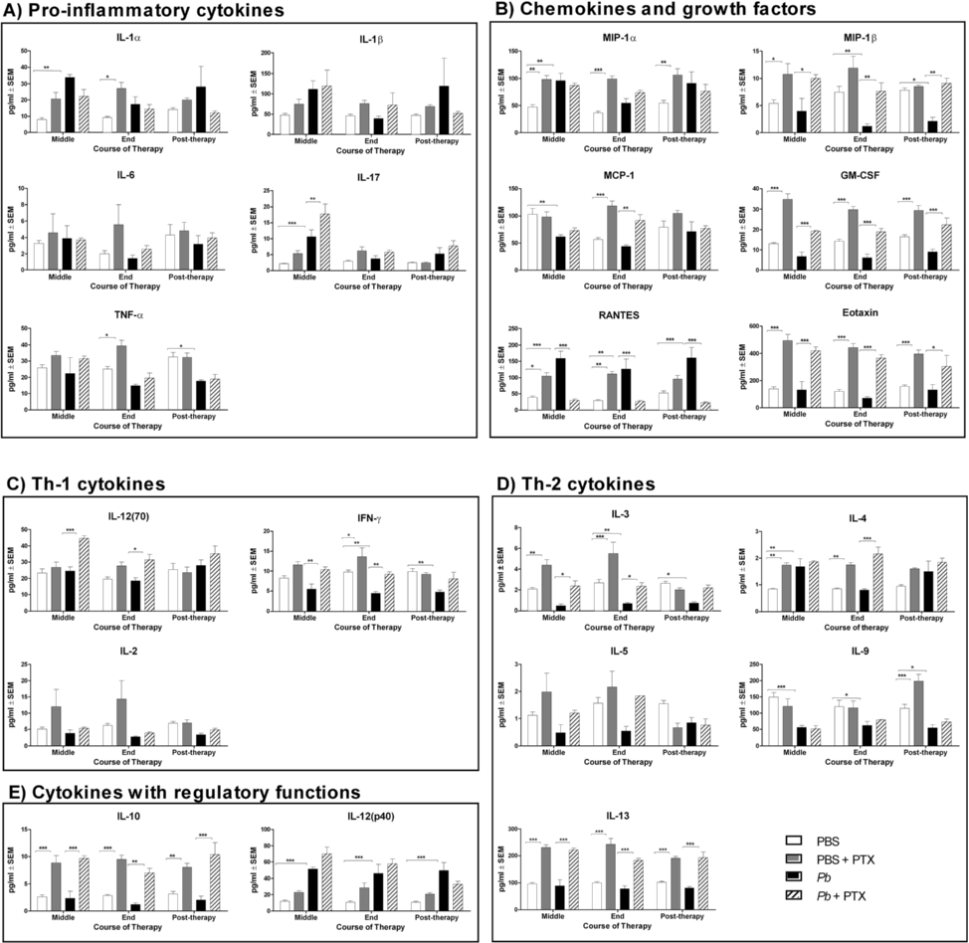
Effects of PTX on pulmonary cytokine levels in healthy and *Pb*-infected mice. The 21 evaluated molecules were grouped into five categories according to their primary functions: **a** pro-inflammatory cytokines, **b** chemokines and growth factors, **c** Th-1 cytokines, **d** Th-2 cytokines, and **e** cytokines with regulatory functions. All molecules were measured in PBS-inoculated mice (*white bars*); PBS-inoculated mice, treated with PTX (*gray bars*); *Pb*-infected mice (*black bars*); and *Pb*-infected mice, treated with PTX (*striped bars*). The graphs show three periods of evaluation corresponding to the middle of the treatment, end of the treatment, and 4 weeks post-treatment (8, 12, and 16 weeks p.i., respectively). The experimental groups were compared using two-way ANOVA with Tukey’s multiple comparison test. The bars show the mean ± SEM. *p <0.05, **p <0.001, ***p <0.0001. *n* = 5 mice per group at each period of evaluation

The administration of PTX to healthy non-infected mice increased MIP1a, granulocyte-monocyte colony-stimulating factor (GM-CSF), regulated upon activation, normal T cell expressed and secreted (RANTES), eotaxin, IL-3, IL-4, IL-13, and IL-10 at the middle and end of the therapy and, in some cases, even post-therapy. *Pb* infection induced the down-regulation of the immune response included reduced expressions of some cytokines during the most chronic periods of infection (8 to 16 weeks). The cytokines included IFN-γ, MIP-1β, TNF-α, IL-3, and IL-9. PTX treatment restored IFN-γ, MIP-1β, and IL-3 levels to normal values (*p <*0.001) (Fig. 3).

PTX therapy in *Pb*-infected mice also increased GM-CSF, IL-12p70, IL-10, IL-13, and eotaxin compared to the levels observed in the *Pb*-infected untreated mice.

RANTES, a chemokine that was up-regulated at all time points post-*Pb* infection, was normalized to the basal levels by PTX treatment.

In the *Pb*-infected mice, the pulmonary levels of IL-1α, IL-1β, IL-2, IL-5, IL-6, IL-9, IL-12 (p40), IL-17, MIP-1α, and TNF-α were not modified by PTX treatment

## Discussion

This study showed that PTX therapy administered at an “early” stage of granulomatous inflammation controlled the progress of the PCM and diminished the pulmonary inflammation and the fungal burden and prevented the appearance of collagen deposits in the pulmonary lesions.

In this study, we applied conventional HRCT to follow the development of the pulmonary lesions in the *Pb*-infected mice and monitored the effects of the therapy. The results of the radiologic evaluations agreed well with the histopathology/histomorphometry; however, in the middle of the therapy (8 weeks p.i), the recognition of the nodules by the radiologists exhibited minimal agreement (*κ* = 0.4) likely because of the small sizes of the nodules (∽400 um). The HU measurements revealed significant decrease in lung density beginning after 4 weeks of treatment (8 weeks p.i) despite the fact that some pulmonary lesions (nodules) were still present at this time. It is possible that the sizes of the nodules did not significantly change in density in the selected regions of interest (ROIs) that were larger (2000 μm in the upper and central regions and 4000 μm in the lower lung regions).

At the end of the treatment with PTX, small infiltrates were observed around the periarterial spaces. It would be interesting for future studies to evaluate whether the inflammatory cells migrated to the lymphatic vessels around the periarterial spaces. PTX led to an improvement in the immune response and reversed the immunosuppression observed in the *Pb*-infected group. The PTX treatment restored the levels of IFN-γ that had been down-regulated by *Pb* infection and increased IL-12p70. These cytokines belong to the Th-1 profile and are recognized for their capacity to accomplish effective responses in PCM [16]. The level of IL-10 was higher in the *Pb*-infected-PTX-treated mice than in the infected controls, which produced high levels of IL-10 but only until 4 weeks p.i. IL-10 is considered to be a multifaceted anti-inflammatory cytokine [17] that helps to control inflammation. The up-regulation of IL-10 by methylxanthines such as PTX has previously been reported [18]. Interestingly, other anti-inflammatory agents, such as steroids and allergen-specific immunotherapy, are also known to elevate endogenous IL-10 levels, which may account for their efficacy [19].

The high levels of IL-10, IL-4, and IL-13 in the *Pb*-infected-PTX-treated mice might explain the persistence of plasmocytes in this group.

RANTES/CCL5 is a potent chemokine for T cells, dendritic cells, eosinophils, NK cells, mast cells, and basophils [20] and is up-regulated at all times post-*Pb* infection in this and other *Pb* infection models [21]. The reduced levels of RANTES observed in the *Pb*-infected-PTX-treated group might be another mechanism by which PTX exerted its immunomodulation in this experimental model.

Interestingly, the PTX treatment induced an eotaxin-rich pulmonary micro-environment. Eotaxin is an eosinophil chemoattractant [22]; however, the numbers of eosinophils did not increase in the lungs of the *Pb*-infected-PTX-treated mice during therapy. Eosinophils appeared after PTX release. One possible explanation is that PTX inhibits the recruitment of cells to the inflammatory sites as suggested by other studies [23–26]. Indeed, PTX has been considered to be a migration-inhibiting factor for human lymphocytes, monocytes, and neutrophils [23–26] and acts by inhibiting the adherence of these cells to the endothelium and their transendothelial migration [27].

It has been increasingly recognized that the inflammatory response and the deregulated cytokine production play key roles in the pathogenesis of many diseases, including certain infectious processes [28]. This study reinforces the idea that, in addition to itraconazole, a second therapeutic avenue for the treatment of PCM might be host-directed therapy with PTX [14]. Host-directed therapy is routinely used for the management of infectious diseases such as pediatric meningitis (corticosteroids and antibiotics) and hepatitis C (IFN-2b and antivirals). However, the concept of correcting the host responses that had been subverted by pathogen virulence strategies has not been fully exploited [29].

Host-directed therapy has been evaluated in experimental PCM. The immunosuppressor cyclophosphamide has been used to modulate immune responses to *Pb* in rats infected with *Pb* yeasts via the intracardiac route [30, 31]. This study reported that treated rats presented with the following: a) a decrease in granuloma size and granulomas with fewer fungal cells, b) a lack of specific antibodies, and c) a significant increase in the paracoccidioidin footpad swelling test (delayed-type hypersensitivity (DTH)). In another study [32], resistant (A/Sn) and susceptible (B10.A) mice were treated with either a low dose of cyclophosphamide or indomethacin, which is a potent inhibitor of prostaglandin synthesis. In the A/Sn mice, the cyclophosphamide induced a recovery of the IgE anti-ovalbumin antibody (OA) antibody response. In the B10.A mice, this effect was extended to IgG1, IgG2a, and total levels of anti-OA antibodies. In general, these studies suggested that “the suppressive stages” in PCM, such as the lack of specific antibodies and DTH, could be inhibited by some immunomodulators such as cyclophosphamide and indomethacin. Indeed, in the present study, we observed that PTX hindered the immune down-regulation induced by *Pb* during the infection’s natural course.

PTX has been studied in other fungal infections previously. Ostrosky-Zeichner et al. [13] reported on the effects of pentoxifylline or dexamethasone alone or in combination with amphotericin-B in experimental mouse cerebral cryptococcosis. The amphotericin-B plus pentoxifylline-treated mice exhibited survived for significantly longer and exhibited decreased fungal burdens in the brain than the mice in the other treated groups [13]. However, in another study, PTX at 20 mg/kg every 8 h had no effect on experimental systemic *Candida albicans* infection, but higher doses of 30 and 60 mg/kg of pentoxifylline every 8 h increased fungal counts in kidneys when compared to the controls [33]. In this last study, the authors used doses that were higher than we employed and administered PTX by intraperitoneal injection, which likely induced an immunosuppressive state.

Granulomas are a hallmark of PCM and tuberculosis and have traditionally been thought to restrict mycobacterial growth. However, analysis of *Mycobacterium marinum* in zebrafish has shown that early granulomas facilitate mycobacterial growth. Uninfected macrophages are recruited to the granuloma where they are productively infected by *M. marinum* to facilitate the disease progress [32]. We believe that a similar situation might occur with *Pb.*

The antifibrogenic effect of PTX that was also observed in this study has been attributed to both the inhibition of extracellular matrix (ECM)-producing cell proliferation and the reduction in the deposition of ECM components, primarily collagen type I, by producer cells [4, 34–37].

The use of PTX apparently caused a paradoxical situation, i.e., simultaneous decreases in the inflammatory reaction and the number of fungi. If PTX induces inhibitions of peripheral blood mononuclear cells, endothelial production of IL-8 and monocyte chemotactic protein-1 (MCP-1), leucocyte chemotaxis and diapedesis, endothelial adhesion molecule expression, T/NK cell cytotoxicity and cytokine production and apoptosis pathways, and the Fas ligand (FasL or CD95L) [4, 34–37], it should be expected to increase the fungal load. However, the opposite was observed. This phenomenon is more puzzling if we consider the possible direct effect of PTX on the fungi; i.e., the inhibition of the *Pb* phosphodiesterase and the consequent increase in intracellular cyclical AMP, which has a role in the dimorphic transition from mycelium to yeast [4, 34–37].

This paradox raises the hypothesis of a possible effect of the inflammatory reaction and its products on fungal viability or a direct and unknown effect of PTX on *P*. brasiliensis; in the first sense and since IFN-γ plays an important role both on the immune response by the modulation of some activities of macrophages and T and B cells and on the host resistance against several pathogens including *P. brasiliensis* [16, 38, 39], it is possible that the increased IFN-γ levels observed in *Pb*-infected-PTX-treated mice could have triggered an effective immune response inducing the elimination of *P. brasiliensis*. In the second sense, some reports have shown that methylxanthines have antifungal properties related to the inhibition of fungal chitinases [40, 41]. More specifically, *Cryptococcus neoformans* and *Aspergillus fumigatus* treated with pentoxifylline exhibited abnormal cell morphology. In addition, pentoxifylline-treated *C. neoformans* showed increased susceptibility to calcofluor and a leaky melanin phenotype consistent with defective cell wall function [40]. Unfortunately, no direct experiments in this sense have been made with *P. brasiliensis*.

Although we found a positive PTX effect on the course of pulmonary lesions in *Pb*-infected mice, our study has some limitations. We focused exclusively on the lungs and other organs which were not included in our study, the evaluation of fungal dissemination, the characterization of the IFN-γ-producing cells, and the treatment of infected IFN-γ knockout mice with PTX to determine whether PTX combats *P. brasiliensis* via the intermediacy of IFN-γ-mediated processes are worthy to be conducted in future studies.

## Conclusions

In conclusion, this study showed that *Pb* infection can be positively modulated by anti-inflammatory therapy with PTX administered at the “early” stages of granulomatous inflammation.

## Methods

### Ethics statement

All animals were handled according to the Colombian national (Law 84 of 1989, Res No. 8430 of 1993) and international (Council of European Communities and Canadian Council of Animal Care, 1998) guidelines for animal research. Additionally, the experimental protocols were approved by the research ethics committee of the Corporación para Investigaciones Biológicas (CIB).

### Mice and experimental groups

BALB/c mice were originally obtained from Taconic Farms, Inc. Quality Laboratory Animals and Services for Research, New York, USA, and the breeding colony was later expanded at the CIB, Medellin, Colombia. The mice were divided into four groups: (A) non-infected or control (*n* = 50), (B) infected with 3 × 10^6^*Pb* conidia (*n* = 50), (C) non-infected-PTX-treated mice (*n* = 30), and (D) *Pb*-infected-PTX-treated mice (*n* = 30).

### Fungus and experimental infection

*Pb* isolate ATCC-60855 from a Colombian patient registered at the American Type Culture (Rockville, MD, USA) and known to produce abundant conidia was used in all experiments [42]. *Pb* conidia were produced and collected as previously described [42–44].

Seven-week-old male mice weighing approximately 20 g were anesthetized by intramuscular injection of a solution containing ketamine hydrochloride (Park, Davis & Company, Berlin, Germany; 100 mg/kg) and xylazine (Bayer, Brazil; 10 mg/kg) [45]. When deep anesthesia was obtained, 3 × 10^6^*Pb* viable conidia (in 0.06 ml of the inoculum) were intranasally (i.n) instilled. Control mice received an intranasal inoculum of 0.06 ml of PBS.

### Pentoxifylline treatment

Pentoxifylline (P1784 Sigma-Aldrich, St. Louis, MO, USA) was provided at a dose of 20 mg/kg in 10 μl of solution. This concentration had been previously used in a mouse model of cryptococcosis [13]. Treatment began 4 weeks p.i when the granulomatous inflammation had begun and continued daily for 8 weeks.

### Evaluation periods

PBS-inoculated and *Pb*-infected mice were evaluated at 0 (2 h), 4, 8, 12, and 16 weeks post-infection. The PTX-treated mice were analyzed during therapy (at 8 and 12 weeks p.i) and 4 weeks post-treatment (at 16 weeks p.i). The chests of ten mice from each group were scanned by HRCT at each period of observation (see section 2.6). Then, the mice were sacrificed by intraperitoneal injection of 1.0 ml of 2.5 % sodium pentothal (Sandoz Laboratories, Kundl, Austria), and their lungs were removed and assigned for further testing in histopathological (five mice) and immunological (five mice) studies.

### High-resolution CT

The thoraxes of the mice were scanned as previously published by our group [46]. Briefly, the mice were anesthetized with ketamine hydrochloride and xylazine and placed in the prone position inside polypropylene tubes (50 ml) that were arranged together in a wood box with parallel holes. All of the animals were placed with their noses in the same vertical plane. Each animal had a code for future identification. The box containing the mice was then placed in the CT gantry for thorax scanning.

CT images were taken in a multislice CT-scanner (General Electric, EU) with 16 canals by applying 140 kV at 165 mAs/s (Kernel U90). Thin-section slices, each 0.625 mm thick and spaced 1 mm apart, covered the complete mouse lung from the apex to the hemidiaphragm. Images were acquired in the axial plane, and the bone algorithm was applied to better visualize the lung. The field of view was 18 cm to simultaneously include all of the animals with a matrix of 512 × 512 and an acquisition time of one second per section. Approximately 20 to 22 slices covered the entire lung.

Following the scan, the mice were placed in their cages to recover from the anesthesia for 35 min. The animals were supplied with standard laboratory diet and water *ad libitum*.

### HRCT-image examination

The image analysis was performed independently and blindly by two radiologists from the Radiology Department of the University Hospital San Vicente de Paul (Medellín, Colombia). The images were visualized using an Advantage workstation version 4.3 (General Electric) via the lung and mediastinal windows.

The pulmonary densities were evaluated as described by Plathow et al. [47] with some modifications [46]. Representative tomographic slides were used to calculate the HU. Briefly, eight ROIs were selected in the following areas of the right and left lungs: the upper or hilar region (approximately 5 slides below the apex, where the main bronchi enter the lungs), the anterior and posterior middle or central region (approximately 12 slides below the apex where the heart presents its largest diameter), and the lower lung region (approximately 18 slides bellow the apex, corresponding to the bases of the lungs). These circles were 2 mm^2^ for the upper and middle regions and 4 mm^2^ for the lower regions.

### Histopathological analysis

After scanning, five animals per group at each period of evaluation were killed by thiopental overdose (Sandoz GmbH., Kundl, Austria; 1 ml at 2.5 %, *i.p.*) in accordance with animal ethical practices. The lungs of the mice were intracardially perfused with 10 % neutral formalin in phosphate-buffered saline, removed, and fixed in the same solution for at least 48 h. The fixed lungs were embedded in paraffin, and coronal sections (4 μm) were stained with the following methods: hematoxylin-eosin (HE) for evaluation of the lung histology and inflammation, Grocott’s methenamine-silver nitrate technique (Grocott) for identification of the fungi, picrosirius with fast green (PIFG) for identification of I and III interstitial collagens [48], Lennert’s Giemsa for lymphocyte and plasma cell recognition [49], sirius red (pH 10.2) for cellular characterization and eosinophil identification [50, 51], and Masson’s trichrome for the collagen fibers and the recognition of the Mott cells that correspond to abnormal plasma cells, and are characterized by the presence of globular cytoplasmic inclusions that are composed of immunoglobulins.

The cellular compositions were evaluated blinded by a pathologist using a semi-quantitative approach. Scores of (−) to (+++) were given according to the degree of infiltration as follows: (+++) for intense, (++) for moderate, (+) for slight, (±) for very slight, and (−) for no reaction [52].

### Quantitative image analysis

The H&E-, Grocott- and PIFG-stained slides were automatically scanned with a ScanScope® CS (Aperio, Vista, CA, USA) at 20X and also analyzed with an Axio Observer.Z1 (Zeiss) coupled with digital camera with AxioCam HRc and software AxioVision 4.7.2 (Zeiss).

The lung inflammatory areas were measured based on one panoramic image of both lungs per mouse. The images were analyzed with the free ImageScope software (http://www.aperio.com/download-imagescope-viewer.asp). The areas of interest (AOIs) corresponding to the inflammatory regions were manually drawn and measured. The percentage of pulmonary area exhibiting an inflammatory reaction was calculated by dividing the sum of the total of the AOIs by the total area occupied by the lung tissue (excluding the air space).

The Aperio positive pixel count algorithm was used to quantify the amount of a specific stain present in a scan slide image. Red pixels in the PIFG-stained slides were measured to quantify collagen, and brown/black pixels in the Grocott’s-stained slides were measured to quantify the glucans present in the fungal wall. The results are expressed as the sum of strong-, medium- and weak-positive pixels in the total area measured.

### Cytokines, chemokines, and growth factor detection by multiplex micro-bead immunoassay

The lungs of five animals per group were individually homogenized in a tissue grinder (Tissue tearor, model 985–370, Biospec Products) with a cocktail solution of protease inhibitors (pepsin 0.1 μM, leupeptin 0.1 μM, phenylmethyl sulfonide fluoride 1 mM, *N*-tosyl-L-phenylalanine chloromethyl ketone 0.2 mM, (a)-p-methyl L-lysine chloromethyl ketone 0.1 mM from Sigma chemical, plus ethylene-diamine-tetraacetic acid (EDTA) 1 nM from Merck Germany). The homogenized lung supernatants were collected by centrifugation at 3000 rpm for 15 min at 4 °C, aliquoted, and stored at −70 °C until the day of analysis.

All homogenized lung supernatants were normalized to 1 mg/ml of protein. Then, a magnetic bead-based multiplex assay containing fluorescent-dyed microspheres conjugated with a monoclonal antibody specific for a target protein was used for cytokines, chemokines, and growth factor measurements according to the manufacturer’s instructions (Bio-Plex pro-mouse cytokine 21-plex assay; Bio-Rad Inc., Hercules, CA, USA). The following molecules were measured: IL1-α, IL-1β, IL-2, IL-3, IL-4, IL-5, IL-6, IL-9, IL-10, IL-12 (p40), IL-12 (p70), IL-13, IL-17, eotaxin, GM-CSF, IFN-γ, monocyte chemoattractive protein (MCP-1/CCL2), macrophage inflammatory protein alpha and beta (MIP-1α/CCL3 and MIP-1β/CCL4), RANTES (CCL5), and TNF-α.

The levels of each molecule were determined in duplicate with the multiplex array reader of the Luminex™ Instrumentation System (Bio-Plex Workstation from Bio-Rad Laboratories). The analyte concentrations were calculated using software provided by the manufacturer (Bio-Plex Manager Software) and are expressed as pg/ml.

### Statistical analyses

The statistical analyses were performed with Prism 5.0 software (GraphPad, San Diego, CA, USA). Non-parametric Kruskal-Wallis tests with Dunn’s post tests were used to compare the selected groups. The values are expressed as medians with interquartile ranges. *p* values under 0.05 were considered statistically significant. The kappa (*κ*) coefficient was used as a statistical measure of agreement between the two radiologists.

## Abbreviations

ECM: extracellular matrix
EDTA: ethylene-diamine-tetraacetic acid
GM-CSF: granulocyte-monocyte colony-stimulating factor
HRCT: high-resolution computed tomography
HU: Hounsfield units
IFN-γ: interferon gamma
IL-3: interleukin 3
MCP-1: monocyte chemoattractive protein 1
MIP-1β: macrophage inflammatory protein-1β
Pb: *Paracoccidioides brasiliensis*
PCM: paracoccidioidomycosis
PMN: polymorphonuclear leukocytes
PTX: pentoxifylline
ROI: region of interest
TNFα: tumor necrosis factor alpha
